# Sinhala translation of the Perinatal Anxiety Screening Scale: a valid and reliable tool to detect anxiety disorders among antenatal women

**DOI:** 10.1186/s12888-020-02757-z

**Published:** 2020-07-21

**Authors:** M. N. Priyadarshanie, M. D. I. A. Waas, C. S. E. Goonewardena, A. Balasuriya, B. C. V. Senaratna, D. M. S. Fernando

**Affiliations:** 1 Department of Nursing & Midwifery, Faculty of Allied Health Sciences, Kotelawala Defence University, Rathmalana, Sri Lanka; 2 Department of Psychiatry, Faculty of Medical Sciences, University of Sri Jayewardenepura, Nugegoda, Sri Lanka; 3 Department of Community Medicine, Faculty of Medical Sciences, University of Sri Jayewardenepura, Nugegoda, Sri Lanka; 4 Department of Public Health, Faculty of Medicine, Kotelawala Defence University, Rathmalana, Sri Lanka; 5 Non-Communicable Diseases Research Centre, University of Sri Jayewardenepura, Nugegoda, Sri Lanka; 6 Department of Physiology, Faculty of Medical Sciences, University of Sri Jayewardenepura, Nugegoda, Sri Lanka

**Keywords:** Perinatal anxiety, Screening, Validation, PASS, Antenatal clinics, Sri Lanka

## Abstract

**Background:**

Anxiety disorders during pregnancy are not routinely assessed in Sri Lanka despite being common and being associated with adverse pregnancy outcomes. Screening can facilitate early detection and management of anxiety and improve pregnancy outcomes. Our aim was to determine the validity of the Sinhala translation of the Perinatal Anxiety Screening Scale (PASS) to detect anxiety among Sri Lankan pregnant women.

**Methods:**

A cross-sectional study was conducted in antenatal clinics of a teaching hospital in Colombo District. The PASS was translated to Sinhala using the standard translation/ back-translation method. Pregnant women (*n* = 221) were sequentially recruited and assessed by a psychiatrist until 81 women with anxiety disorder were diagnosed using the International Classification of Diseases-10 criteria (gold standard). The Sinhala translation of the PASS (PASS-S) was administered to all recruited women, including 140 women without anxiety. Receiver-Operating- Characteristic (ROC) analysis was performed, the optimal cut-off score for PASS-S was determined, and its validity was assessed using sensitivity, specificity, predictive values and positive and negative likelihood ratios. Internal consistency was assessed using Cronbach’s alpha. Test-retest and inter-rater reliability for PASS-S score and anxiety classification were assessed using intra class correlation coefficient (ICC) and Cohen’s kappa (k), respectively.

**Results:**

The mean age (±SD) of women was 30(±5.8) years, and 53.7% were multiparous. A psychiatrist diagnosed anxiety disorder was made in 37.0% of women, while the PASS-S, at its optimal cut-off of ≥20, classified 37.5% of women as having anxiety disorders. The area under the ROC curve for the PASS-S was 0.96 (95%CI 0.94–0.99). Sensitivity, specificity and positive and negative predictive values of the PASS-S were 0.93 (95% CI 0.84–0.97), 0.90 (95% CI 0.83–0.94), 0.85 (95% CI 0.75–0.90) and 0.95 (95% CI 0.89–0.98), respectively. Positive and negative likelihood ratios were 8.8 (95% CI 5.3–14.5) and 0.08 (95%CI 0.04–0.18), respectively, and the internal consistency was high (Cronbach’s alpha 0.95). Four-factor structures obtained by exploratory factor analysis were “acute anxiety and adjustment”, “social anxiety, specific fears and trauma”, “perfectionism and control” and “general anxiety”.Test-retest reliability was high for the PASS-S score (ICC 0.85[95% CI 0.65–0.96]) and anxiety classification (k 0.77[95% CI 0.34–1.2]). Inter-interviewer reliability was also high (ICC 0.92[95% CI 0.81–0.97] for the PASS-S score and (k0.86 [95% CI 0.59–1.1] for anxiety classification).

**Conclusion:**

The Sinhala translation of the PASS is a valid and reliable instrument to screen for anxiety disorders among antenatal women in Sri Lanka.

## Background

Anxiety symptoms are common during pregnancy and the postpartum period [1], which could be due to continuation or worsening of pre-existing anxiety, caused by the physiological and psycho-social changes associated with pregnancy or new onset anxiety; both of which are likely to be higher among perinatal women than in the general population [2–4]. Anxiety during pregnancy may be generalised anxiety or pregnancy-specific anxiety. Pregnancy-specific anxiety is an emotional state resulting from the anticipated uncertainties related to pregnancy-specific issues during antenatal and/or post-partum periods, especially due to worries about labour, wellness of the baby to be born and neonatal care [5, 6].

Anxiety during pregnancy in low- and middle-income countries [7] is as high as 25%, compared to 10% in developed countries [8]. The prevalence of antenatal anxiety and depression is 20% in Asian women [9] but the prevalence of antenatal anxiety is not known to Sri Lankans. However, antenatal anxiety often coexists with antenatal depression and is a strong predictor of postpartum depression. In Sri Lanka the latter two conditions have high prevalence of 16% [10] and 27% [11], respectively, which suggests that undetected antenatal anxiety among Sri Lankan women is likely to be high.

Limited evidence suggests that antenatal anxiety is associated with adverse foetal and maternal outcomes including still birth, intrauterine growth restriction (IUGR), prolonged labour, caesarean deliveries, preterm birth, low birth-weight, and low Apgar scores at birth [5, 12–15]. Early detection and management of antenatal anxiety will improve pregnancy outcomes [16]. The gold standard to detect anxiety is diagnosis by a psychiatrist [17]. However, as it is not feasible for all pregnant women to be routinely assessed by a psychiatrist, standard questionnaires are used to screen pregnant women for anxiety. Some of these questionnaires screen for only general anxiety states (e.g. State-Trait Anxiety Inventory [STAI] [18]) or generalized anxiety disorder (e.g.Generalized Anxiety Disorder 7-item [GAD-7] scale [19]), while others screen for only pregnancy-specific anxiety (e.g.Pregnancy related Anxiety Questionnaire [PrAQ] [20] and Pregnancy Anxiety Scale [21]). Using only the pregnancy-specific anxiety questionnaires, focusing on symptoms such as fears related to pregnancy and child birth, the mother’s concerns on her physical appearance and relationship issues within the family, is useful to capture the anxiety related to pregnancy itself, but has limited utility in detecting a broader range of anxiety disorders [22]. Questionnaires that screen for a spectrum of anxiety disorders during pregnancy will be more useful to screen antenatal women, as it is the presence of any anxiety disorder that is likely to cause adverse outcomes, rather than any specific anxiety disorder including anxiety specific to pregnancy.

The Perinatal Anxiety Screening Scale (PASS) is an acceptable, psychometrically-sound questionnaire that has performed well in screening for antenatal and postnatal anxiety disorders [22] including those due to pregnancy. It screens for a broad range of anxiety presentations, and it is not confounded by the physiological symptoms of pregnancy. Being the first tool developed to screen a broad range of anxiety disorders among a perinatal population, originally validated among antenatal and postnatal women, the PASS possesses a valid and reliable case finding ability [22]. It has been utilized in many countries to detect antenatal anxiety disorders [23–26]. Good validity and reliability reported for translations such as the PASS-Turkish version (PASS-TR) [24] and the PASS Bangladesh version (Bangala PASS) [25] suggest that it is robust in cross-cultural adaptation.

In Sri Lanka, the prevalence of adverse pregnancy outcomes remains high despite other maternal health indices being satisfactory. This could be due to causes that are not assessed routinely during pregnancy, including anxiety disorders. However, no valid screening tool is available in Sri Lanka to detect antenatal anxiety disorders. Although the Edinburgh Postnatal Depression Scale (EPDS) which screens for postnatal depression has been used [27], this includes only three items to screen for anxiety. Furthermore, these items do not distinguish between anxiety and depression [2]. Therefore, a valid and reliable questionnaire to screen for antenatal anxiety disorder in Sri Lanka is a timely need. Our aim was to translate the PASS into Sinhala language and validate it among pregnant women to address this requirement.

## Methods

### Study design and setting

This was a descriptive cross-sectional study conducted among antenatal women attending hospital antenatal clinics (ANCs). The study was conducted in the ANCs of Colombo South Teaching Hospital (CSTH), which is the second largest tertiary-care institution in Colombo District with a bed strength of 1096 and an annual turnover of 150,000 in-ward patients and 75,000 out-patients. Antenatal women from its catchment area, which is urban/semi-urban, and those referred to it from elsewhere due to risk conditions are registered at ANCs run by three obstetrics units, and they are followed up until the delivery [28, 29].

### Study population and sampling

The study population was pregnant women who attend ANCs of CSTH.

Pregnant women attending ANCs at CSTH were recruited to the study irrespective of the trimester of pregnancy (first, second or third trimesters). The inclusion criterion was age of at least 18 years. Those with disabilities such as hearing difficulty, visual and speaking problems were excluded. Although diagnosed but currently untreated mental disorders were also an exclusion criterion, no one was excluded based on this criterion.

As per the expected sensitivity (70%) and specificity (30%) of the PASS [22], the calculated sample size was 81 antenatal women with anxiety disorder and 81 women without anxiety disorder.

Eligible pregnant women were recruited using systematic random sampling with a sampling interval of 5. The sampling frame was the list of registered women in the ANC attendance registry.

### Data collection procedure

Participants were given written information detailing the purpose of the study, voluntary participation, procedures and time required for the procedures, potential benefits, discomforts, confidentiality and the ability to terminate participation at participant’s discretion without having to face any adverse consequences. The information was also read out by the Principal Investigator (PI), and queries raised by the participants were clarified. The participants were given an additional 1 h on the average to ask any further questions. The decision to participate in the study was purely voluntary and written informed consent was taken from all participants. The participants were also given contact details of the research team to enable them to clarify any concerns even after the study participation was over.

The PI, who is a graduate nurse, checked the eligibility for the study based on the selection criteria by directly questioning the participants and checking the antenatal records. Recruited women were assessed by a psychiatrist as described below. Recruitment continued until 81 women with anxiety disorder were diagnosed.

### Measures

#### Screening test: perinatal anxiety screening scale

The PASS is a 31-item validated instrument used to screen for anxiety disorders and cover all domains of anxiety in antenatal and postpartum women [22]. Each item enquires about the presence of anxiety symptoms during the preceding 1 month and is scored on a Likert scale ranging from 0 (not at all) to 3 (almost always). A total score between 0 and 20 is considered as “asymptomatic”, 21–41 as “mild-moderate anxiety symptoms” and 42–93 as “severe anxiety symptoms”. An overall score higher than 26 indicates a high risk of presenting with anxiety disorders [22].

#### Reference test: diagnostic interview using International Classification of Diseases-10

The International Classification of Diseases and Related Health Problems (ICD-10) provides clinical descriptions and diagnostic guidelines of diseases. It also provides the inclusion and exclusion criteria for diagnoses, allowing a degree of flexibility to be retained for diagnostic decisions in clinical setting [30]. Diagnostic interview by a psychiatrist, as per ICD-10 criteria, is the gold standard of diagnosis of anxiety disorder.

The present study was conducted in three phases. Firstly, the PASS was translated into Sinhala and its content, conceptual and semantic validity were assessed. Secondly, the construct validity of the Sinhala translation was examined. Thirdly, the criterion validity and reliability of the Sinhala translation was determined.

### Translation of PASS into Sinhala language

**Fig. 1:**
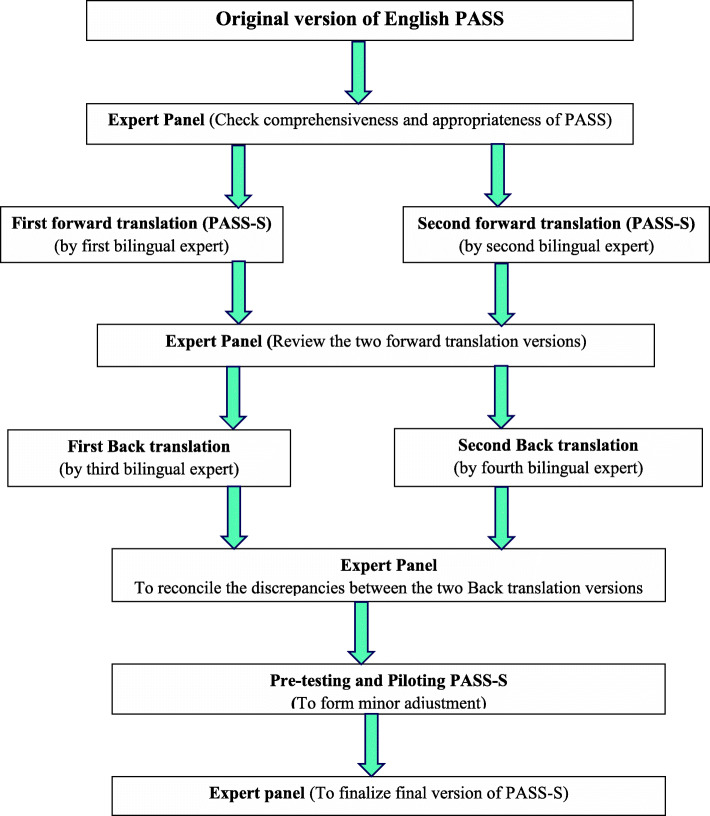
Translation procedure of PASS-S - Sinhala translation of PASS

Translation and adaptation was carried out according to the World Health Organization (WHO) guidelines on the process of translation and adaptation of instruments [31]. Stages of translation of PASS (Fig. 1) are given below.

Firstly, the English version of PASS was reviewed by a local expert panel to determine its comprehensiveness and appropriateness for local use. This panel consisted of three psychiatrists, an obstetrician, three community physicians and two clinical psychologists. All 31 items of PASS were marked as suitable for screening anxiety disorders in antenatal women by this expert panel. Secondly, two independent bi-lingual experts translated the PASS into Sinhala. The two Sinhala versions were compared by an expert panel, which included a psychiatrist and three psychologists, and the inadequate terminologies/conceptions of the translation and any discrepancies between two Sinhala translation versions were resolved.

This first draft of the Sinhala version was back-translated into English by two other independent bi-lingual translators who were blinded to the English PASS. The back-translation was reviewed by an expert panel, which included a psychiatrist and three psychologists, and 28 items were rated as the correct translation, and the other three items, as needing minor revisions. Discrepancies were discussed and resolved. This panel provided consensual approval on the preservation of original English meaning of all items of PASS.

The standard translation-back translation method that was followed in the study was similar to how the translation of PASS was done in Bangladesh [25] and in Turkey [24].

This Sinhala version was pretested among 20 women 18 years of age or older, attending ANCs of another major tertiary care hospital in Colombo District, which is similar in setting and patients to the CSTH, to assess comprehensibility of the questions. Every fifth woman, as per the clinic registration number, was recruited until 20 women were recruited. They were interviewed by the PI using the probing method in cognitive interviewing approach to confirm the clarity of the questionnaire, understanding of the language used and consistency of the answers given.

Upon pre-test, the final Sinhala translation of PASS (PASS-S) was checked by an expert panel comprising three psychiatrists, who were not involved in the initial translation process, for its content, conceptual and semantic validity. The final expert panel confirmed all 31 items of the PASS-S to be of appropriate standard to screen for anxiety symptoms of antenatal women in Sri Lanka, of which 30 items were rated as culturally relevant, and one item, as moderately relevant.

The PASS-S was piloted among 20 women 18 years of age or older, attending primary-care ANCs at community level, to identify any practical constraints in administering this in such clinics where any antenatal screening is likely to take place, including the convenience of administration, participants’ problems when responding and any other issues. Those who participated in the pre-test and the pilot study were not included in the actual validation study. Although the sample selected for the pilot test was from different ANCs, they were similar in their characteristics to the sample used for the validation study. All procedures conduced in the main study were piloted except for the clinical interview conducted by the psychiatrist. No practical constraints were encountered and the feasibility of implementation of the validation study was confirmed.

### Construct validity of Sinhala translation of PASS

The construct validity of PASS-S was assessed using the methods described under the section on statistical analysis presented below.

### Criterion validity of Sinhala translation of PASS

Validation of PASS-S against the gold standard was conducted among pregnant women attending ANCs at CSTH.

Recruited women were first assessed by a psychiatrist using ICD-10 criteria. A total of 221 women were recruited including 81 with anxiety disorders. This was immediately followed by administration of the PASS-S as an interviewer administered questionnaire by two trained nurses. The time taken to complete an interview using the PASS-S was 6–8 min on average.

The findings of the psychiatrist and the nurses were blinded to each other. The PASS-S was re-administered to a randomly-selected 20 women 2–3 days after the initial assessment by the same nurse who administered the PASS-S at the first interview. To another randomly-selected 20 women, the PASS-S was re-administered 2–3 days after the initial assessment by a nurse different from the one who administered the first interview.

### Statistical analysis

Descriptive data on participants are reported as numbers and percentages or means and standard deviations (SDs). To examine the construct validity of PASS-S, sample adequacy and suitability for factor analysis were conducted using Kaiser-Meyer-Olkin (KMO) test and Bartlett’s test, respectively. Principal component analysis (PCA) for factor extraction in exploratory factor analysis (EFA) and oblique rotation method which was suggested for correlated variables [32] were used.

Confirmatory factor analysis (CFA) was also performed after ensuring that the required assumptions were fulfilled. LISREL 10.2 software was used for this. Additivity test was employed to check whether the scale was an additive scale. A four-factor model was evaluated. The indices used to determine the model fit included the absolute fit indices, relative fit indices and parsimony fit indices. Absolute fit indices were chi-squared test, root mean squared error of approximation (RMSEA), goodness of fit index (GFI), adjusted goodness of fit index (AGFI) and standardized root mean squared residual (SRMR). Relative fit indices were comparative fit index (CFI) and non-normed fit index (NNFI), while parsimony fit indices were parsimony goodness of fit index (PGFI) and parsimonious normed fit index (PNFI).

RMSEA values below 0.05 indicate a good fit to data, values between 0.05 and 0.08, an acceptable fit, values between 0.08 and 0.10, a marginal fit and values above 0.10, a poor fit [33, 34]. For the CFI and NNFI, values above 0.95 [35] indicate a good fit to data while for GFI and AGFI, over 0.90 indicate a good fit [36].

Using the psychiatrist’s diagnosis as the gold standard, the receiver-operating-characteristic (ROC) curve was graphed for the total scores for PASS-S obtained by participants. The optimal cut-off score of the PASS-S for detecting anxiety in pregnancy was determined using Youden’s index [37]. Performance of the PASS-S at this optimal cut-off score against the psychiatrist’s diagnosis was assessed using sensitivity, specificity and predictive values.

Internal consistency of the PASS-S was measured using Cronbach’s alpha. Test-retest reliability and inter-rater reliability of the total PASS-S scores were assessed using intra-class correlation coefficient (ICC) for the total PASS-S score, and those of anxiety classification (symptomatic of anxiety disorder/ not symptomatic) were assessed using Cohen’s kappa (k).

## Results

The mean age ± SD of the recruited antenatal women was 30 ± 5.8 years. Most (74.2%) of the participants had completed primary education and 56.1% were multiparous (Table 1). Nearly half (48.9%) were in the third trimester of pregnancy, while 36.7 and 14.5% were in the second and first trimesters respectively.

**Table 1:** Basic characteristics of the antenatal women (*n* = 221)

Variable	Frequency	Percentage (%)
**Age (Years)**		
18–23	30	13.6
24–29	80	36.2
30–35	75	33.9
36–41	36	16.3
**Level of Education**		
No schooling	1	0.5
Primary	164	74.2
Secondary	56	25.3
**Monthly income Level (SLR)**		
0–20,000	33	14.9
20,001–40,000	118	53.4
40,001–60,000	42	19
60,001–80,000	0	0.0
80,001–100,000	18	8.1
100,001 and above	10	4.5
**Pregnancy and childbirth status**		
First pregnancy	97	43.9
Living child 1	94	42.5
Living children 2	23	10.4
Living Children 3	6	2.7
Living Children 4	0	0.0
Living Children 5	1	0.5
**Trimester in current pregnancy**		
Trimester1	32	14.5
Trimester 2	31	36.6
Trimester 3	108	48.9

The psychiatrist diagnosed 37.0% of women (*n* = 81) as having anxiety disorder using ICD-10 Criteria. Most women with anxiety had generalized anxiety disorder or phobias (Table 2).

**Table 2:** Severity and domains of anxiety as assessed by gold-standard (psychiatrist using ICD-10 criteria)

Severity of anxiety	Frequency	Percentage (%)
Mild	47	58.02
Moderate	31	38.27
Severe	03	3.71
**Domains of anxiety**		
Generalized anxiety disorder	30	37.03
OCD	02	2.46
Phobias (blood and injection)	32	39.5
Social phobia	06	7.4
Specific fear (Child birth)	04	4.93
PTSD	01	1.23
Panic disorder	06	7.4

### Criterion validity of PASS-S

**Fig. 2:**
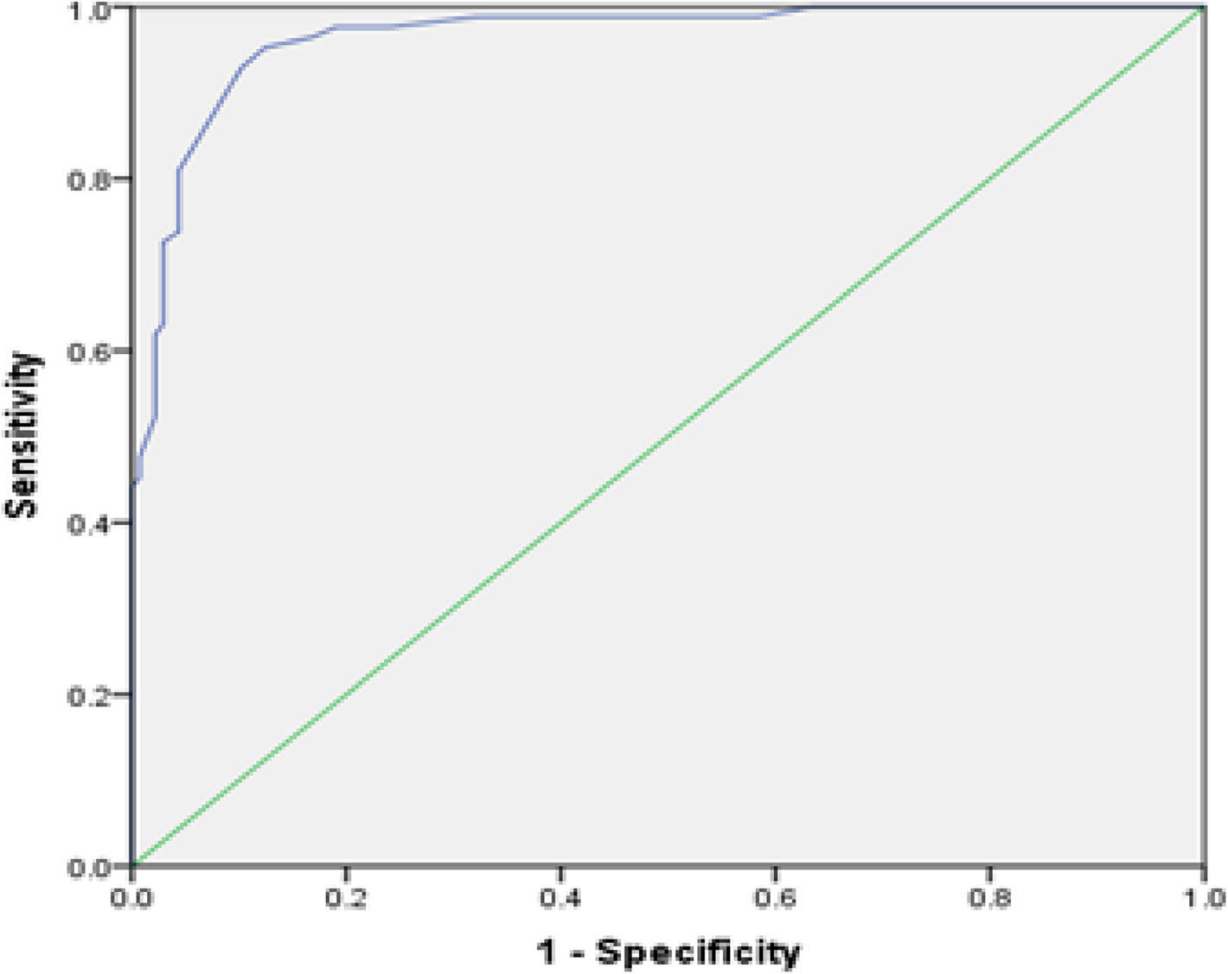
ROC Curve-The receiver operating characteristic curve of the participants – Area under the curve: 0.96

The area under the ROC curve for PASS-S scores was 0.96 (95% CI: 0.94–0.99) (Fig. 2). The optimal sensitivity and specificity for the PASS-S was found at the score of ≥20. At this cut-off score, the sensitivity was 0.93 (95% CI: 0.84–0.97) and the specificity was 0.90 (95% CI: 0.83–0.94) (Table 3). The positive and negative predictive values of PASS-S at this cut-off score were 0.85 (95% CI: 0.75–0.90) and 0.95 (95% CI: 0.89–0.98) respectively. The positive and negative likelihood ratios were 8.8 (95% CI: 5.34–14.5) and 0.08 (95% CI: 0.04–0.18), respectively.

**Table 3:** Sensitivity and specificity of PASS-S at different cut-off scores (only the scores immediately above and below the optimal cut-off score are shown)

PASS-S Score	Sensitivity	Specificity
12.50	.988	.569
13.50	.988	.613
14.50	.988	.679
15.50	.976	.759
16.50	.976	.810
17.50	.964	.832
18.50	.952	.876
**19.50**	**.929**	**.898**
20.50	.869	.927
21.50	.810	.956
22.50	.738	.956
23.50	.726	.971
24.50	.631	.971
25.50	.619	.978

According to PASS-S scores, 3.7% of the pregnant women (*n* = 3) had severe anxiety symptoms, while 96.3% (*n* = 79) had mild to moderate anxiety symptoms. Cronbach’s alpha for internal consistency was 0.95 (95% CI: 0.93–0.96). The ICC for total PASS-S score was 0.85 (95% CI: 0.65–0.96) for test-retest reliability and 0.92 (95% CI: 0.81–0.97) for inter-rater reliability. The Cohen’s kappa for anxiety classification (symptomatic of anxiety disorder/ not symptomatic) was 0.77 (95% CI: 0.34–1.2) for test-retest reliability and 0.86 (95% CI: 0.59–1.1) for inter-rater reliability.

### Construct validity of PASS-S

The KMO test value for sampling adequacy was 0.95. Bartlett’s test of sphericity was significant (χ^2^ = 4390.838; df 496; *p* < 0.01).

As per the four-factor model analysis, the total variance was 58.1% (*n* = 221). An examination of the factor loadings after rotation (Table 4) showed that factor 1 (“acute anxiety and adjustment”) accounted for 46.9% of this total variance. Out of the remaining 11.2% of the total variance, factor 2 (“social anxiety, specific fears and trauma”) accounted for 4.0%, factor 3 (“perfectionism and control”), for 3.9%, and factor 4 (“general anxiety”), for 3.3%. These four factors’ factor loading described the characteristics of 31 variables included in the PASS-S.

**Table 4:** Factor Structure of PASS-S

Factors	Factor			
	1	2	3	4
**1. “Acute anxiety and adjustment”**				
25. Losing track of time and can’t remember what happened	.775			
27. Anxiety getting in the way of being able to do things	.754			
29. Fear of losing control	.751			
30. Feeling panicky	.744			
28. Racing thoughts making it hard to concentrate	.688			
26. Difficulty adjusting to recent changes	.684			
23. Avoiding things which concern me	.658			
24. Feeling detached like you’re watching yourself in a movie	.632			
21. Feeling really uneasy in crowds	.543			
22. Avoiding social activities because I might be nervous	.440			
7. Really strong fears about things, eg needles, blood, birth, pain, etc	.428			
31. Feeling agitated	.413		.-367	
**2.** “**Social anxiety, specific fears and trauma”**				
19. Worry that I will embarrass myself in front of others		.719		
1. Worry about the baby/pregnancy		.611		
4. Worry about many things		.602		
20. Fear that others will judge me negatively		.487		
5. Worry about the future		.407		.301
18. Upset about repeated memories, dreams or nightmares		.421		
3. A sense of dread that something bad is going to happen	.334	.364		
17. Being ‘on guard’ or needing to watch out for things	.301	.319		
**3. “Perfectionism &control**”				
11. Having to do things in a certain way or order			−.587	
12. Wanting things to be perfect	.324		−.560	
13. Needing to be in control of things			−.703	
14. Difficulty stopping checking or doing things over and over			−.645	
15. Feeling jumpy or easily startled			−.596	
16. Concerns about repeated thoughts			−.646	
**4. “General anxiety”**				
6. Feeling overwhelmed				.726
10. Difficulty sleeping even when I have the chance to sleep				.534
2. Fear that harm will come to the baby			−.336	.509
9. Repetitive thoughts that are difficult to stop or control				.399
8. Sudden rushes of extreme fear or discomfort				.465

The results of EFA indicated that four-factor arrangement in PASS-S (with Eigen values over 1 and suppressing absolute value less than .03) explains all the factors included in PASS-S.

### Confirmatory factor analysis

Fit indices obtained in the CFA indicated that the data fit the hypothesized measurement model perfectly. The four-factor model gave a χ^2^ value of 705.57 (df 428; *p* < 0.001). RMSEA was 0.056 while SRMR, GFI and AGFI were 0.046, 0.83 and 0.803 respectively. CFI, NNFI, PGFI and PNFI were 0.92, 0.919, 0.716 and 0.766 respectively. The four-factor model was closer to the good fit indices in each absolute, relative, and parsimony fit indices.

## Discussion

The Sinhala translation of the PASS has produced a valid and a reliable tool to assess anxiety disorder during pregnancy in Sri Lanka. The PASS-S showed high sensitivity, specificity, and predictive values in a local clinic setting. Its test-retest and inter-rater reliability measures were also high. It also showed good content, conceptual and semantic validity similar to those in the two studies conducted in Turkey [24] and Bangladesh [25], indicating appropriateness of the PASS for use in multicultural settings.

Sampling adequacy for EFA was excellent (KMO = 0.95) and was similar to the English PASS validation study (KMO = 0.96) [22]. Inter-item correlation was sufficiently large for PCA (Bartlett’s test of sphericity, *p* < 0.00), and was also similar to that of the English PASS. Although the number of factors identified was 4 in the English PASS, the item distribution for the factors slightly varied from the English PASS and PASS-TR. In the English PASS, factor 1 (acute anxiety and adjustment) had items that addressed symptoms of panic disorder, dissociative disorder and adjustment difficulties, factor 2 (general worry and specific fears) covered symptoms of general anxiety disorder and phobia, factor 3 (perfectionism, control and trauma) included symptoms of obsessive compulsive disorder and post-traumatic stress disorder, and factor 4 (social anxiety) had questions to determine social anxiety. However, in the PASS-S, factor direction and the titles of factors were altered. Factors 1–4 were reclassified as “acute anxiety and adjustment” covering symptoms of panic disorders, acute stress and adjustment disorders, “social anxiety, specific fears and trauma” covering symptoms of social anxiety disorder, phobias and post-traumatic stress disorder; “perfectionism and control” covering symptoms of obsessive compulsive disorder and “general anxiety” covering symptoms of generalized anxiety disorder, respectively. These changes were essential components of intercultural adaptation, and the factor structure of the current study reflects specific classification of anxiety disorder in the Sri Lankan context. The factor analysis showed that generalized and acute anxiety symptoms are more prominent (factors loaded under factor 1 of the factor structure) among antenatal women in Sri Lanka while excessive worry and specific fears were prominent anxieties among participants of the English PASS validation study [22].

The optimal cut-off point of ≥20 for PASS-S was lower than the score of 26 for the English PASS [22] but higher than the score of 16 for the PASS-TR [24]. It suggests that Sri Lankan women’s risk for anxiety disorders occurs at a lower symptoms threshold compared with their Australian counterparts but at a higher threshold compared with Turkish women [24]. The specificity of the PASS-S was similar to that of the PASS-TR but higher than that of the English PASS while the sensitivity of the PASS-S was higher than those of both the English PASS [22] and the PASS-TR [24]. One reason for comparable sensitivity with the PASS-TR may be due to assessment of criterion validity in both these studies against the gold standard and using the entire sample. In the English validation study, criterion validity was assessed only in a sub-sample. The socio-cultural factors that differentially affect participants from different geographical areas may also have contributed to the variable perception and reporting of anxiety symptoms by participants in different studies. The Bangladesh study did not report sensitivity, specificity or cut-off scores since criterion validity was not assessed [25]. When a screening tool has high sensitivity and high specificity, it detects large proportions of true positives and true negatives, and its quality is at an optimum level [38]. Furthermore, the accuracy of a screening test depends on its ability to differentiate between those who have and who do not have the disease and is measured by the area under the ROC curve. The PASS-S accurately classified 96% of women with and without anxiety disorder compared to 93% for the PASS-TR [24] and 70% for the English PASS [22]. The minimal misclassification that occurred when PASS-S was used makes it an ideal tool to be used in settings where the gold standard assessment is not feasible.

The internal consistency of the PASS-S was similar to the Turkish PASS [24], the Bangla PASS [25] and the English PASS [22]. The high test-retest ICC for the total score (0.85) and Cohen’s kappa (0.77) for anxiety disorder classification indicates that the symptoms measured in the PASS-S were stable over time. A high test-retest reliability for the total score was also seen in the English PASS (Pearson *r* = 0.74 [22]) and the Bangla PASS (Pearson *r* = 0.83 [25]). Our study is the first to report inter-interviewer reliability for the PASS in any language and showed that this was high for the total score (ICC 0.92) and anxiety disorder classification (Cohen’s kappa 0.86). This suggests that the PASS-S is suitable for use in clinic settings that often have multiple interviewers.

The PASS-S was administered as an interviewer-administered questionnaire to maximize the response rate and to ensure that literacy level of the participants did not influence the responses [39, 40]. The time taken to complete it was 6–8 min on average. This was not very different from the time taken to complete the English PASS and the PASS-TR (2–10 min), which were self-administered questionnaires [22, 24].

The main strength of our study is that the PASS-S was validated against the gold standard of a psychiatric diagnosis of anxiety disorders. Ours is only the second study to do this, the first being validation of the PASS-TR against both Diagnostic and Statistical Manual of Mental Disorders-4th edition (DSM-IV) criteria and ICD-10 criteria [24]. Furthermore, the performance of the PASS–S compared well with the English PASS and its other translated versions for validity, reliability and discriminatory ability. One limitation in our study is that our sample represented mostly the urban and semi-urban antenatal women in Sri Lanka. Although nearly half of the Sri Lankan women belong to this category [41], rural women might perceive and express symptoms of anxiety disorders differently [42]. In this context, the PASS-S may need further validation before using it to screen antenatal women of a rural population.

The good validity and reliability of the PASS-S make it a suitable tool to be used in clinical practice and in research, and may even be used routinely to screen for anxiety disorders among antenatal women in Sri Lanka. Further studies to assess the feasibility of using the PASS-S as a screening tool to detect anxiety disorders during routine antenatal care are recommended. Validation of the PASS in Tamil, the other local language in Sri Lanka, is also recommended.

## Conclusion

The PASS-S is a valid and reliable instrument to assess anxiety during pregnancy among Sinhala speaking women, and may be useful in routine assessment of anxiety in such women.

## Supplementary information

**Additional file 1: Table S1**. Result of content/consensual validity-Appropriateness of factor structure use in Sri Lankan context. **Table S2**. Comparison of Factor Structure in PASS and PASS-S. **Table S3**. Fit indices, their description and cut-off values used for interpreting model fit in CFA (1).

## Data Availability

The datasets used and/or analyzed (those including personal information of antenatal women) during the current study are available from the corresponding author on reasonable request.

## Abbreviations

PASS: Perinatal Anxiety Screening Scale
PASS-S: Sinhala version of Perinatal Anxiety Screening Scale
STAI: State-Trait Anxiety Inventory
GAD-7: Generalized Anxiety Disorder 7-item
EPDS: Edinburgh Postnatal Depression Scale
PASS-TR: Turkish translation of Perinatal Anxiety Screening Scale
WHO: World Health Organization
ROC: Receiver-operating-characteristic
PI: Principal Investigator
ERC: Ethics Review Committee
k: Cohen kappa
ICC: Intra-class correlation coefficient
EFA: Exploratory factor analysis
PCA: Principal component analysis
CFA: Confirmatory factor analysis
ANC: Antenatal clinics
ICD-10: International Classification of Diseases-10
RMSEA: Root mean square error of approximation
RMR: Root mean square Residual
GFI: Goodness of fit index
AGFI: Adjusted goodness-of-fit index
CFI: Comparative fit index
KMO: Kaiser-Meyer-Olkin
DSM-IV: Diagnostic and Statistical Manual of Mental Disorders, 4th edition
