# Supplementary material for: Sinhala translation of the Perinatal Anxiety Screening Scale: a valid and reliable tool to detect anxiety disorders among antenatal women
Source: BMC Psychiatry. 2020 Jul 21;20:381. doi: 10.1186/s12888-020-02757-z (PMC7374883; doi:10.1186/s12888-020-02757-z)
Supplement: Supplementary file 1 — Additional file 1: Table S1. Result of content/consensual validity-Appropriateness of factor structure use in Sri Lankan context. Table S2. Comparison of Factor Structure in PASS and PASS-S. Table S3. Fit indices, their description and cut-off values used for interpreting model fit in CFA (1). [file 12888_2020_2757_MOESM1_ESM.pdf]

Table S1-Result of content/consensual validity-Appropriateness of factor structure use in Sri Lankan context

Factor structure of PASS	Appropriateness of Use in Sri Lankan Context
**1.“Excessive worry and specific fear”**	
1. Worry about the baby/pregnancy	Appropriate Standards
2. Fear that harm will come to the baby	Appropriate Standards
3. A sense of dread that something bad is going to happen	Appropriate Standards
4. Worry about many things	Appropriate Standards
5. Worry about the future	Appropriate Standards
6. Feeling overwhelmed	Appropriate Standards
7. Really strong fears about things, eg needles, blood, birth, pain, etc	Appropriate Standards
8. Sudden rushes of extreme fear or discomfort	Appropriate Standards
9. Repetitive thoughts that are difficult to stop or control	Appropriate Standards
10. Difficulty sleeping even when I have the chance to sleep	Appropriate Standards
**2. “Perfectionism, control and trauma”**	
11. Having to do things in a certain way or order	Appropriate Standards
12. Wanting things to be perfect	Appropriate Standards
13. Needing to be in control of things	Appropriate Standards
14. Difficulty stopping checking or doing things over and over	Appropriate Standards
15. Feeling jumpy or easily startled	Appropriate Standards
16. Concerns about repeated thoughts	Appropriate Standards
17. Being 'on guard' or needing to watch out for things	Appropriate Standards
18. Upset about repeated memories, dreams or nightmares	Appropriate Standards
**3. “Social anxiety”**	
19. Worry that I will embarrass myself in front of others	Appropriate Standards
20. Fear that others will judge me negatively	Appropriate Standards
21. Feeling really uneasy in crowds	Appropriate Standards
22. Avoiding social activities because I might be nervous	Appropriate Standards
23. Avoiding things which concern me	Appropriate Standards
**4. “Acute anxiety and adjustment”**	
24. Feeling detached like you're watching yourself in a movie	Moderately Appropriate Standards
25. Losing track of time and can't remember what happened	Appropriate Standards
26. Difficulty adjusting to recent changes	Appropriate Standards
27. Anxiety getting in the way of being able to do things	Appropriate Standards
28. Racing thoughts making it hard to concentrate	Appropriate Standards
29. Fear of losing control	Appropriate Standards
30. Feeling panicky	Appropriate Standards
31. Feeling agitated	Appropriate Standards
	Appropriate Standards

Table S2-Comparison of Factor Structure in PASS and PASS-S

Factor structure of PASS	Factor structure of PASS-S
**1.“Excessive worry and specific fear”**	**1. Acute anxiety and adjustment**
1. Worry about the baby/pregnancy	25. Losing track of time and can't remember what happened
2. Fear that harm will come to the baby	27. Anxiety getting in the way of being able to do things
3. A sense of dread that something bad is going to happen	29. Fear of losing control
4. Worry about many things	30. Feeling panicky
5. Worry about the future	28. Racing thoughts making it hard to concentrate
6. Feeling overwhelmed	26. Difficulty adjusting to recent changes
7. Really strong fears about things, eg needles, blood, birth, pain, etc	23. Avoiding things which concern me
8. Sudden rushes of extreme fear or discomfort	24. Feeling detached like you're watching yourself in a movie
9. Repetitive thoughts that are difficult to stop or control	21. Feeling really uneasy in crowds
10. Difficulty sleeping even when I have the chance to sleep	22. Avoiding social activities because I might be nervous
**2.”Perfectionism,control and trauma”**	31. Feeling agitated
11. Having to do things in a certain way or order	7. Really strong fears about things, eg needles, blood, birth, pain, etc
12. Wanting things to be perfect	
	**2.“Social anxiety, specific fears & trauma”**
13. Needing to be in control of things	19. Worry that I will embarrass myself in front of others
14. Difficulty stopping checking or doing things over and over	
15. Feeling jumpy or easily startled	1. Worry about the baby/pregnancy
16. Concerns about repeated thoughts	4. Worry about many things
17. Being 'on guard' or needing to watch out for things	20. Fear that others will judge me negatively
18. Upset about repeated memories, dreams or nightmares	5. Worry about the future
**3.”Social anxiety”**	18. Upset about repeated memories, dreams or nightmares
19. Worry that I will embarrass myself in front of others	3. A sense of dread that something bad is going to happen
20. Fear that others will judge me negatively	17. Being 'on guard' or needing to watch out for things
21. Feeling really uneasy in crowds	**3. “Perfectionism & control”**
22. Avoiding social activities because I might be nervous	11. Having to do things in a certain way or order
23. Avoiding things which concern me	12. Wanting things to be perfect
**4. “Acute anxiety and adjustment”**	13. Needing to be in control of things

24. Feeling detached like you're watching yourself in a movie	14. Difficulty stopping checking or doing things over and over
25. Losing track of time and can't remember what happened	15. Feeling jumpy or easily startled
26. Difficulty adjusting to recent changes	16. Concerns about repeated thoughts
27. Anxiety getting in the way of being able to do things	**4. “General anxiety”**
28. Racing thoughts making it hard to concentrate	6. Feeling overwhelmed
29. Fear of losing control	10. Difficulty sleeping even when I have the chance to sleep
30. Feeling panicky	2. Fear that harm will come to the baby
31. Feeling agitated	9. Repetitive thoughts that are difficult to stop or control
	8. Sudden rushes of extreme fear or discomfort

Table S3-Fit indices, their description and cut-off values used for interpreting model fit in CFA (1)

Fit Index	Description	Cut-off values
**<u>Absolute fit indices</u>**		
1. Chi-Square test	Assess the magnitude of discrepancy between the sample and fitted covariance matrices	p > 0.05
2. RMSEA	Indicates the extent to which the model, with unknown but optimally chosen parameter estimates would fit the population’s covariance matrix	0.08 – 0.10: Mediocre model fit< 0.08: Good model fit
3. GFI	Calculates the proportion of variance that is accounted for by the estimated population covariance	> 0.90 : Good model fit
4. AGFI	Adjust the GFI based upon degrees of freedom	> 0.90 : Good model fit
5. SRMR	Indicates the square root of the difference between the residuals of the sample covariance matrix and the hypothesized covariance model	< 0.05: Good model fit
**<u>Relative fit indices</u>**		
1. NNFI	Assess the model by comparing the chi value of the model to the chi value of the null model	> 0.95 : Good model fit
2. CFI	A revised form of the NFI which takes into account the sample size(Byrne, 1998)	> 0.95 : Good model fit
**<u>Parsimony fit indices</u>**		
1. PGFI	Based upon the GFI by adjusting for loss of degrees of freedom	>0.5/no absolute threshold values
2. PNFI	Based upon the GFI by adjusting for loss of degrees of freedom	>0.5/no absolute threshold Values

1. Wickramasinghe ND, Dissanayake DS, Abeywardena GS. Validity and reliability of the Maslach Burnout Inventory-Student Survey in Sri Lanka. BMC Psychol. 2018 Nov 12;6(1):52.

PASS-Sinhala version (PASS-S)

පූර්ව ප්‍රසව මානසික කාංසාව පරීක්ෂා කිරීම

පූර්ව ප්‍රසව................................ පසු ප්‍රසව ....දිනය ....

ගර්භයට සති ගණන කියද? .... දරුවාගේ වයස..............................

ඔබ පසුගිය මාසය තුල කොපමණ වතාවක් පහත සඳහන් දේ අත්දැක තිබේද? සෑම ප්‍රශ්නයටම ඔබගේ අත්දැකීමට ඉතාම සමීප පිළිතුර ඉදිරියේ (√) ලකුණක් යොදාන්න .

	කොහෙත්ම නැත	සමහර වෙලාවට ඇත	බොහෝ විට	හැමදාම සැමවේලාවෙම
01. දැරුවා ගැන හෝ ගැබ් ගැනීම පිළිබඳව ඇති කණස්සල්ලය.				
02. දරුවාට හානියක් වේදෝයි කියන බය				
03. යම් කිසි නරක දෙයක් සිදු වේය කියන බිය				
04. බෙහෝ දේවල් ගැන කණස්සල්ලයක් ඇතිවීම				
05. අනාගතය ගැන කණස්සල්ලයක් ඇතිවීම.				
06. තමාට අවශ්‍ය දේවල් කරගැනීමට නොහැකි යැයි යන හැගීම.				
07. යම් යම් දේ පිළිබඳව දැඩි බියක් දැනීම. උදාහරණ:- ඉඳිකටු, ලේ, දරු උපත, වේදනාව යනාදිය				
08. හදිසියේ ඇති වන දැඩි බිය හෝ අපහසුතාව.				
09. පාලනය කිරීමට හෝ නවත්වා ගැනීමට අපහසු ලෙස නැවත නැවත මතුවන සිතුවිලි				
10. නින්දට අවස්ථාව ලද විට දී පවා නිදා ගැනීමට අපහසු වීම.				
11. යම්කිසි ක්‍රමයකට හෝ පිළිවෙළකට සමහර දේ සිදු කිරීමට සිදුවීම.				
12. සියල්ලම සර්ව සම්පූර්ණව සිදුවිය යුතුයැයි සිතීම.				
13. සියළු දේ තමන්ට අවශ්‍ය ලෙස පාලනය කර ගැනීමට ඕනෑවීම				

14. නවත්වා ගැනීමට අපහසු ලෙස එකම දේ නැවත නැවත කිරීම හෝ නැවත නැවත පරීක්ෂා කර බැලීම				
15. ඉක්මණින් කලබලයට හෝ තිගැස්සීමට ලක්වීම.				
16. යළි යළිත් මතුවන සිතුවිලි පිළිබඳ කණස්සලු ගතියක් ඇතිවීම.				
17. නිතරම ආරක්ෂා සහගත ලෙස කටයුතු කිරීම හෝ අනතුරු/කරදර පිළිබඳ නිතර අවධානයෙන් සිටීම .				
18. යළි යළිත් මතකයට නැගෙන මතකයන්, සිහින, හීනෙන් බයවීම, අධික තැතිගැන්වෙන සිහින සම්බන්ධව කණස්සල්ලයට පත්වීම.				
19. අනෙක් අය ඉදිරියේ තමන් විසින් තමන්වම ලජ්ජාවට පත්කර ගනී යැයි කණස්සල්ලයක් ඇති වීම.				
20. අනෙක් අය තමන්ව අසුභවාදී ව හෝ අඩුපාඩු සහිත ලෙස විනිශ්චය කරාවි යන බිය.				
21. සෙනගක් ඉදිරියේ සිටින විට දැඩි අපහසුවක් දැනීම.				
22. තමන්ව කලබලයට හෝ මානසික අපහසුවට පත්වේදෝයි යන බිය නිසා සමාජ ක්‍රියා කාරකම් වලින් වැලකී සිටීම.				
23. තමන්ව අවධානයට ලක් කරවන දේවල් වලින් ඉවත්ව සිටීම.				
24. ඔබ ඔබව චිත්‍රපටයක දකින සේ වියුක්ත හැගීමක් ඇති වීම.				
25. වෙලාව යන බව නොදැනීම සහ යම් යම් වේලාවන් වල දී කුමක් සිදුවී ද යන්න අමතකවී යාම.				
26. මෑතකදී සිදුවූ වෙනස් වීම් වලට අනුගත වීමට නොහැකි වීම.				
27. සිතේ ඇති කලබල ගතිය හෝ බය ගතිය නිසා අවශ්‍ය දේවල් කර ගැනීමට බැරි වීම .				
28. වේගයෙන් එහෙ මෙහෙ යන සිතුවිලි නිසා සිත එකතැන් කරගැනීම අපහසු වීම.				
29. තමාව පාලනය කරගැනීමට නොහැකි වේ යැයි යන හය.				
30. අධික තැතිගැනීමක් ඇතිවීම.				
31. අධික කළබලකාරී සිතක් ඇතිවීම.				
